# EHEC O111:H8 strain and norovirus GII.4 Sydney [P16] causing an outbreak in a daycare center, Brazil, 2019

**DOI:** 10.1186/s12866-021-02161-x

**Published:** 2021-03-29

**Authors:** Liliana Cruz Spano, Caroline Gastaldi Guerrieri, Lays Paula Bondi Volpini, Ricardo Pinto Schuenck, Jaqueline Pegoretti Goulart, Elizabeth Boina, Celia Regina Nascimento Recco, Rodrigo Ribeiro-Rodrigues, Luís Fernando dos Santos, Tulio Machado Fumian

**Affiliations:** 1 Department of Pathology, Health Sciences Center, Federal University of Espírito Santo, Vitória, Brazil; 2 State Health Secretariat, Central Public Health Laboratory, Vitoria, Espírito Santo Brazil; 3 Municipal Health Secretariat, Epidemiology Service, Vila Velha, Espírito Santo Brazil; 4 Adolfo Lutz Institute, Centre of Bacteriology, National Reference Laboratory for Escherichia coli Enteric Infections, São Paulo, Brazil; 5 Laboratory of Comparative and Environmental Virology, Oswaldo Cruz Institute, Fiocruz, Rio de Janeiro, Brazil

**Keywords:** Enterohemorrhagic *Escherichia coli*, Shiga-toxigenic *Escherichia coli*, Norovirus, Outbreaks, Hemolytic uremic syndrome, Child daycare center

## Abstract

**Background:**

This study describes the investigation of an outbreak of diarrhea, hemorrhagic colitis (HC), and hemolytic uremic syndrome (HUS) at a daycare center in southeastern Brazil, involving fourteen children, six staff members, six family members, and one nurse. All bacterial and viral pathogens detected were genetically characterized.

**Results:**

Two isolates of a strain of enterohemorrhagic *Escherichia coli* (EHEC) serotype O111:H8 were recovered, one implicated in a case of HUS and the other in a case of uncomplicated diarrhea. These isolates had a clonal relationship of 94% and carried the *stx2a* and *eae* virulence genes and the OI-122 pathogenicity island. The EHEC strain was determined to be a single-locus variant of sequence type (ST) 327. EHEC isolates were resistant to ofloxacin, doxycycline, tetracycline, ampicillin, and trimethoprim-sulfamethoxazole and intermediately resistant to levofloxacin and ciprofloxacin. Rotavirus was not detected in any samples, and norovirus was detected in 46.7% (14/30) of the stool samples, three of which were from asymptomatic staff members. The noroviruses were classified as the recombinant GII.4 Sydney [P16] by gene sequencing.

**Conclusion:**

In this outbreak, it was possible to identify an uncommon *stx2a* + EHEC O111:H8 strain, and the most recent pandemic norovirus strain GII.4 Sydney [P16]. Our findings reinforce the need for surveillance and diagnosis of multiple enteric pathogens by public health authorities, especially during outbreaks.

**Supplementary Information:**

The online version contains supplementary material available at 10.1186/s12866-021-02161-x.

## Background

Enterohemorrhagic/Shiga toxin-producing *Escherichia coli* (EHEC/STEC) is an important human pathogen that has been responsible for several outbreaks of hemorrhagic colitis (HC) and hemolytic uremic syndrome (HUS), but in Brazil, the frequency of EHEC/STEC infection is low [1]. Several serotypes with different somatic (O) and flagellar (H) antigens are described for EHEC/STEC, internationally the most commonly reported serotypes are O157:H7 (prototype strain), O26:H11, O45:H2, O103:H2, O111:H8(NM), O121:H19, and O145:NM [2–4]. EHEC/STEC O111:H8 is the most common serotype circulating in Brazil, although the occurrence of outbreaks attributed to this or other EHEC/STEC serotypes is uncommon in the country [5]. This may be due to the cross-reaction of antibodies for enteropathogenic *E. coli* strains (EPEC) with similar EHEC/STEC strains, hindering infection by the latter [6, 7].

EHEC/STEC infection is considered to be associated with the consumption of meat and unpasteurized dairy products contaminated with cattle excrement during harvesting or processing. These animals are recognized as important hosts of O157 and non-O157 [8–11]. Pathogenesis depends on the production of phage-encoded Shiga toxins (Stx1 and/or Stx2) and their subtypes, which disrupt protein synthesis in endothelial cells, leading to vascular injury, especially in the kidney, brain, gut, and pancreas [12]. The severity of infection in human hosts is strongly correlated with toxin subtype. Infections caused by strains secreting Stx2a and/or Stx2c are more often associated with an unfavorable prognosis [13]. A low frequency of severe cases is observed in Brazil, which could partially be explained by the high frequency of isolates carrying only Stx1 and the low frequency of carriage of the Locus of Enterocyte Effacement (LEE) [5].

The LEE is a large chromosomal pathogenicity island that encodes genes for intimate bacterial adherence to the intestinal epithelium, resulting in the formation of attaching and effacing lesion (A/E) and diarrhea [14]. The presence of the LEE is typically determined by detection of the gene *eae*, which is essential to A/E adhesion. LEE is a definitive virulence factor of enteropathogenic *E. coli*, but it is also present in a subset of STEC strains, which are often termed EHEC [14]. EHEC strains are associated with a greater risk of severe disease, than *eae*^−^ STEC [15]. Additionally, there are several other non-LEE virulence genes believed to enhance the pathogenic potential of both *eae*^+^ and *eae*^−^ STEC strains [16, 17].

Of all bacterial and viral infectious agents of acute gastroenteritis, noroviruses are the most common cause of outbreaks worldwide and, unlike EHEC/STEC, are frequently detected in Brazil [18]. In addition to the low infectious dose, several other factors favor the spread of norovirus, such as its high environmental stability, excretion by asymptomatic individuals, and the high viral load shed in feces and vomit. Long-lasting immunity is not achieved, given that mutational events and recombination are common, ensuring infection in all age groups [19, 20].

Noroviruses are small nonenveloped icosahedral viruses consisting of single-stranded RNA with the open reading frames ORF1, ORF2, and ORF3, which encode nonstructural proteins, major (VP1) capsid proteins, and minor (VP2) capsid proteins, respectively [21]. The ORF1–ORF2 junction is the hotspot of recombination events, allowing classification of noroviruses on the basis of both ORFs. For instance, RNA-dependent RNA polymerase typing is used to segregate noroviruses into 10 P-groups and 62 P-types [22], whereas VP1 nucleotide sequencing allows classification into 10 genogroups (GI to GX) and 49 genotypes [21–23]. Noroviruses belonging to GI, GII, and GIV are known to infect humans [22]. Norovirus GII.4 is the most commonly reported cause of infections, and, since the mid-1990s, pandemic variants have emerged, namely US 95–96, Farmington Hills 2002, Hunter 2004, Den Haag 2006, New Orleans 2009, and Sydney 2012 [20]. GII.4 Sydney emerged in 2012 and has been circulating worldwide since then [24]. Recently, a new variant (GII.4 Hong Kong) was reported, although its circulation has been limited to Eurasia since mid-2017 [25].

A recent outbreak of diarrhea, HC, and HUS occurred at a daycare center in southeastern Brazil involving children, staff, and family members. Considering the severity of manifestations and the rarity of HUS outbreaks in Brazil, we investigated the possibility of both bacterial and viral agents as potential causes.

## Results

**Fig. 1:**
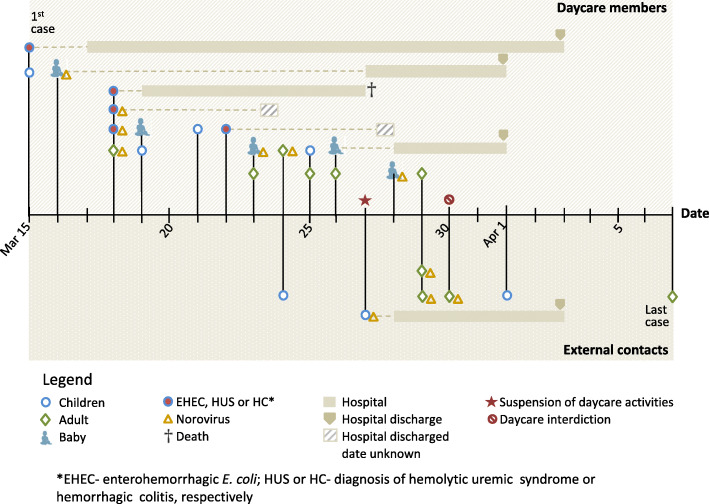
Timeline of cases involved in the outbreak. Onset of diarrhea in children and adults, hospitalization cases, enterohemorrhagic *Escherichia coli* (EHEC)-positive cases and/or diagnosis of hemolytic uremic syndrome (HUS) or hemorrhagic colitis (HC), and norovirus-positive cases. Cases that occurred among daycare staff members and external contacts are shown above and below the horizontal line, respectively

An outbreak of gastroenteritis occurred in Vila Velha, Espírito Santo State, Brazil, from March 15 to April 7, 2019. The primary case of childhood diarrhea occurred 8 days after the daycare center resumed its activities following the carnival holiday. Secondary cases emerged and were recorded in the following days (Fig. 1).

A total of 123 children were enrolled in the daycare center, distributed in classes according to their age (Table 1). Symptomatic children were from four of the seven classes. The outbreak affected 27 individuals, including fourteen children, six staff members, six family members, and one nurse.

**Table 1:** Distribution of symptomatic cases among children, stratified by daycare center class

Class^a^	Total no. of children	No. (%) of affected children	Symptomatic cases		
			Severe cases^b^	EHEC-positive^d^	Norovirus-positive
Baby	12	5 (41.7%)	2	0	3
1	16	0 (0)	0	0	0
2	20	5 (25%)	3	2	1
3A	19	1 (5.3%)	1	0	1
3B	19	1 (5.3%)	0	0	0
4	18	0 (0)	0	0	0
5	19	2^c^ (10.5%)	0	0	0
Total	123	14	6	2	5

^a^Distributed according to age. ^b^Children diagnosed with hemolytic uremic syndrome, hemorrhagic colitis, or diarrhea. ^c^Norovirus was not investigated in one case. ^d^Enterohemorrhagic *Escherichia coli* (EHEC) was isolated from a child with hemolytic uremic syndrome and a child with diarrhea. Staff members from classes 2 and 3A were also affected

Six of the 14 symptomatic children developed severe symptoms and required hospitalization; three of them had HUS, one had HC, and two had diarrhea only. The primary case was among the severe cases (Fig. 1). One child died on March 27, after 8 days of hospitalization (Fig. 1), having attended daycare for 1 day only (March 15) after the carnival holiday. It was reported that, a week before, the child, a classmate (the primary case with HC), and their respective families went to the beach together, where they ingested fried fish, shrimp, and coconut water. No other family members displayed symptoms, nor were there other severe cases of HC or HUS outside the daycare. Clinical data are presented in Table 2 and the Supplementary Table.

**Table 2:** Clinical data of symptomatic patients with severe manifestations, enterohemorrhagic *Escherichia coli* infection, or norovirus infection

Clinical data	Cases, ***n*** (%) (***n*** = 27)	EHEC/HUS/HC^**a**^, ***n*** (***n*** = 5)	Norovirus, ***n*** (%) (***n*** = 11)
Diarrhea	26 (96.3)	5	11 (100)
Mucus in stool	5 (18.5)	4	2 (18.2)
Blood in stool	5 (18.5)	4	2 (18.2)
Vomiting	12 (44.4)	3	6 (54.5)
Fever	11 (40.7)	2	0
Convulsion	1 (3.7)	1	0
Hematuria	2 (7.4)	2	4 (27.3)
Hospitalization	7^b^ (25.9)	4	4 (27.3)

^a^*EHEC* enterohemorrhagic *E. coli* isolates (*n* = 2); *HUS* hemolytic uremic syndrome, *HC* hemorrhagic colitis. ^b^One child was positive for both EHEC and norovirus

Diarrheal cases among staff members occurred between March 18 and 29 (Fig. 1). The affected teachers and assistants were from classes 2 and 3A; other symptomatic cases occurred in general service workers. Four external cases occurred in a single family (son, husband, and parents of a teacher). The last external case of diarrhea occurred in a nurse (April 7) that cared for the child with HUS, who died (March 27) (Fig. 1).

Daycare activities that might have favored the dissemination of infectious agents were provision of handmade burgers to children (March 11), recreational activities in a wading pool shared by older children and young children wearing diapers (March 12 and 14), and a picnic in the classroom (March 15).

All daycare activities were voluntarily suspended on March 27, and the center was interdicted by health authorities on March 30 upon identification of EHEC.

Two *stx2*^+^ *eae*^+^ *E. coli* isolates were recovered; both were serotyped as O111:H8 and possessed the virulence genes *stx2a* and *eae* γ2, allowing their identification as EHEC. Analysis for additional virulence markers revealed the presence of the genes *efa1*, *nleE*, *nleB*, and *sen*. The clonal relationship between isolates, determined by pulsed-field gel electrophoresis (PFGE) with *Xba*I restriction enzyme, was 94%, characterizing them as the same strain. Multilocus sequence typing (MLST) of the EHEC O111:H8 isolates identified alleles 6, 1483, 4, 85, 43, 12, and 7 of *adk*, *fumC*, *gyrB*, *icd*, *mdh*, *purA*, and *recA* genes, respectively. The allelic profile varied only in the *fumC* gene in relation to sequence type (ST) 327, being therefore characterized as a single-locus variant (SLV) of this ST. The ST number of the isolates recovered in this outbreak was not registered, as the Enterobase no longer accepts Sanger sequencing.

The EHEC O111:H8 isolates were recovered from two patients who had not received antibiotic treatment, one with HUS and one with uncomplicated diarrhea. The other patients with HC or HUS received antibiotic treatment, and no bacteria were isolated from their samples. Both EHEC isolates were resistant to ofloxacin, doxycycline, tetracycline, ampicillin, and trimethoprim-sulfamethoxazole and intermediately resistant to levofloxacin and ciprofloxacin. No *Shigella* spp. or *Salmonella* spp. were identified.

All specimens were negative for rotavirus and norovirus GI. Norovirus GII was detected in 46.7% (14/30) of the analyzed stool samples, of which 11 (78.6%) were from symptomatic individuals, including one child diagnosed with HUS/HC, one child with EHEC, and two staff members. Six asymptomatic staff members were included in the study, and three of them tested positive for norovirus GII. Whereas EHEC and HC/HUS occurred during the first week and only in children from the daycare center, norovirus GII was detected up to the end of the outbreak and in all age groups (Fig. 1).

**Fig. 2:**
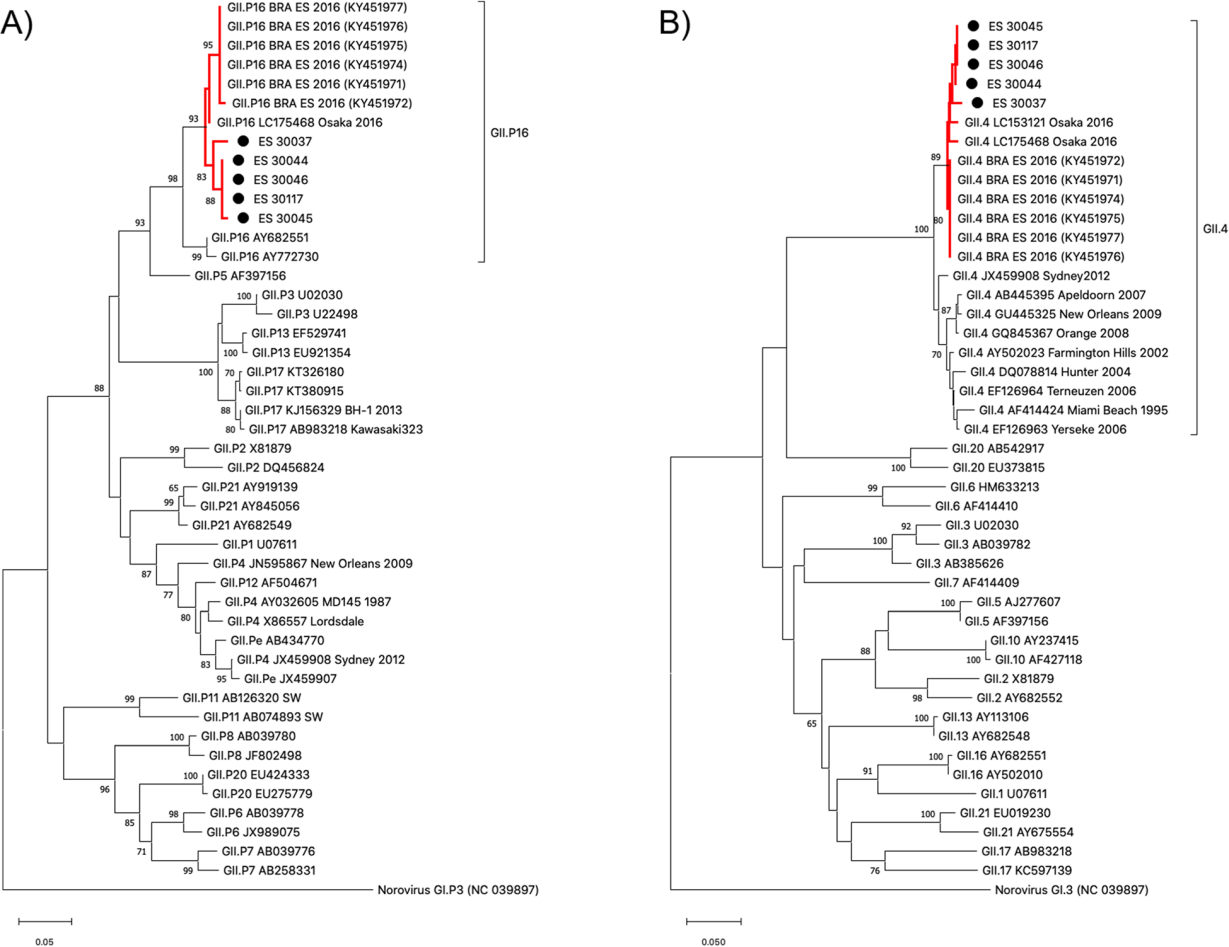
Phylogenetic tree based on (**a**) partial polymerase and (**b**) capsid regions of norovirus GII. Norovirus strains isolated from patients during the outbreak are shown in this phylogenetic analysis and are marked with a black filled circle. Branches containing strains classified within the cluster of the emergent genotype GII.4 Sydney [P16] are highlighted in red on both trees. Reference strains were downloaded from GenBank and labeled with the genotype followed by their accession number. Sequence alignments were performed using the MUSCLE algorithm. Neighbor-joining phylogenetic trees were constructed using MEGAX software with bootstrap tests (2000 replicates) based on the Kimura two-parameter model. Bootstrap percentage values are shown at each branch point for values ≥60%

Five norovirus samples were sequenced and classified as the recombinant GII.4 Sydney [P16]. The strains showed nucleotide similarities ranging from 98.2 to 100%. Phylogenetic analysis of the norovirus genome revealed that all outbreak isolates clustered within the recently emergent GII.4 Sydney [P16] recombinant genotype (reference strain LC175468 Osaka 2016) and were genetically related to other Brazilian strains isolated in Espírito Santo State in 2016, belonging to the same emergent recombinant genotype (Fig. 2).

## Discussion

In this paper, we describe a gastroenteritis outbreak associated with both EHEC and norovirus GII infection that occurred at a daycare center, that also involved external contacts. The occurrence of severe cases of diarrhea with HC and HUS during the first week of the outbreak initially masked the concomitant outbreak caused by norovirus, whose presence was confirmed in the following weeks.

EHEC O111:H8 was isolated from a child with uncomplicated diarrhea and from one of the four cases diagnosed with HC or HUS. Some of these children were undergoing antimicrobial treatment prior to sample collection, which may have affected the probability of bacterial pathogen isolation. Although O111:H8 is one of the most common EHEC/STEC serotypes in Brazil and worldwide [5], this is the first isolate of EHEC O111:H8 carrying *stx2a* as the sole *stx* gene identified in Brazil, and, to the best of our knowledge, there has been only a single report of a similar isolate, in Japan [26]. The SLV of ST327 in EHEC O111:H8 observed in this study contrasts with the EHEC O111:H8 *stx2a*^+^ strain identified in Japan, characterized as ST16 [26]. Interestingly, Cavalcanti et al. [5] showed that ST16 predominates in O111:H8 strains circulating in Brazil. However, of the several atypical EPEC O111:H8 Brazilian strains, it is noteworthy that three were ST327 (L.F. Santos, unpub. data). This suggests that, among atypical EPEC O111:H8, a subgroup of ST327 strains may be more permissive to the Stx2a phage and that the isolates from this outbreak might have been derived from one of these strains. Although the ST of the outbreak strain has not been defined, it is known to be a SLV of ST327, and possibly they form a clonal group.

We identified the *efa1*, *nleE*, *nleB*, and *sen* genes amongst the virulence markers of the OI-122 pathogenicity island. Of note, plasmid-encoded virulence markers such as *katP*, *ehxA*, and *espP*, which are commonly found in EHEC O111:H8, were absent from the two isolates, as were genetic markers encoding other toxins and adhesins of diarrheagenic *E. coli*. We believe that *stx2a* and OI-122 genes, recognized as markers of highly virulent strains for humans [27], might have enhanced the virulence of the EHEC O111:H8 strain.

Norovirus genotype GII.4 Sydney [P16] was the other pathogen detected in the daycare center outbreak. This genotype was the most recent recombinant norovirus strain derived from the previous GII.4 variant (GII.4 Sydney), harboring a new polymerase type [P16] [24, 28]. GII.4 Sydney [P16] was previously detected in our geographic area in 2015 and 2016, shortly after its first description in Asia [28, 29]. Other studies have demonstrated the continuous spread of this emergent strain throughout the world [30, 31].

The highest rate of norovirus infection occurred in the baby class of the daycare center. The first case was also detected in the baby class, whereas secondary cases occurred in two children, one from class 2 and the other from class 3A. It is possible that the spread was limited by the interruption of daycare activities. Asymptomatic cases among children might have occurred, but, unfortunately, it was not possible to collect samples from all children and staff.

A similar outbreak caused by both EHEC and norovirus was previously described at a Japanese kindergarten involving children, staff, and family members; Stx1-producing EHEC O26:H11 and norovirus GII were detected among the cases [32]. Likewise, a large outbreak of norovirus GII in Australia following a dinner event included one individual also infected with STEC O128:H2 *stx1*^+^ [33].

There have been studies identifying HUS as a possible complication of norovirus infection [34–36]. Single adult cases with underlying conditions were reported by Sugimoto et al. [34] and Gaur et al. [35]. In contrast, Daher et al. [36] described HUS in a healthy 9-month-old male infant admitted to a hospital with acute viral gastroenteritis symptoms. Norovirus was the only infectious agent detected among several other bacterial and viral agents investigated. However, as EHEC is a well-established causative agent of HUS, we believe it was the most likely cause of HUS in the outbreak described in the present study.

Identifying the source of infection was a challenge, mainly because the primary case was not promptly reported to health authorities or daycare center staff. Diarrhea is not an uncommon event in daycare centers, which may lead to initial disregard of cases. Therefore, it was not possible to track the source of infection. Food and water samples collected at the daycare center tested negative for bacterial enteric pathogens (R.R. Rodrigues, pers. comm.). Unfortunately, in the present case, the disease involved the highly virulent agent EHEC as well as norovirus, the main infectious agent of childhood hospitalization for gastroenteritis in countries with national rotavirus vaccination programs, such as Brazil [18, 37].

Considering that the first symptomatic case caused by norovirus occurred the day after the primary HC case, it is possible that both EHEC and norovirus pathogens could have been simultaneously introduced at the daycare center. However, it is more likely that there were two different sources of infection. This hypothesis is supported by the observations that HC symptoms were first identified in a child from class 2 (norovirus-negative) and that the first child to present norovirus (EHEC-negative) was from the baby class.

We highlight that three staff members infected with norovirus, including one who worked in the kitchen, reported no symptoms of acute diarrhea. Indeed, prolonged viral excretion may occur after symptomatic infection and among asymptomatic individuals [19]. Susceptibility to symptomatic infection by noroviruses is largely dependent on histo-blood group antigen and secretor status [38, 39]. We hypothesize that conditions in the daycare center were favorable for environmental contamination and person-to-person spread of both the virus and bacterium.

In conclusion, our study reports a rare and severe diarrhea outbreak in Brazil caused by EHEC and the latest recombinant GII.4 Sydney [P16] norovirus. We emphasize the emergence of the uncommon EHEC O111:H8 serotype with an unusual ST, carrying only the *stx2a* toxin gene, in addition to *eae* and markers of the OI-122 pathogenicity island, giving rise to a remarkably virulent clone. Our findings reinforce the need for surveillance and diagnosis of multiple enteric pathogens by public health authorities, particularly during outbreaks.

## Method

### Cases and clinical data

This is a descriptive cross-sectional study investigating an outbreak of gastroenteritis in a daycare center in Vila Velha, Espírito Santo State, Brazil. The investigation began on March 22, 2019, the date of the first notification of the outbreak to the municipal health authorities of Vila Velha. Clinical and epidemiological data were obtained by health agents. Sample collection started on March 23 and finished on April 10.

Thirty-three individuals were included in the study. Stool samples were obtained from 32 of them for bacterial (*n* = 32) and viral (*n* = 30) investigation. A total of 27 cases of diarrhea and/or vomiting occurred in the daycare center, affecting fourteen children, six staff members (teachers, assistants, and general service workers), six family members (three children and three adults), and one nurse. Six asymptomatic staff members were also included in this study. The criteria for inclusion of asymptomatic staff were as follows: being a food handler or working in a class with a large number of severe cases, such as HC or HUS (classes 2 and 3A).

The daycare center was closed for 30 days starting from March 27. Diarrhea was defined as loose stools occurring at least three times a day. The primary case was defined as the one that appeared without known direct contact with other patients, and secondary cases as those that arose more than 24 h after the onset of the primary case.

### Ethical aspects

Epidemiological data were obtained from the Espírito Santo State Central Laboratory, Vitória, through an authorization term for database use.

This study was approved by the Research Ethics Committee of the Health Sciences Center of the Federal University of Espírito Santo (Protocol no. 3.584.448, September 18, 2019), with a waiver of informed consent, in accordance with Brazilian Resolution on Human Research no. 466 (Certificate of Presentation for Ethical Appreciation no. 20181519.7.0000.5060). All methods were carried out in accordance with relevant guidelines and regulations.

### Bacterial isolation, identification of *E. coli* pathotypes, EHEC serotyping, and antimicrobial susceptibility testing

Stool samples from 32 individuals were transported in Cary–Blair transport medium to the state public health laboratory for bacterial isolation on MacConkey (Basingstoke, UK) and Hektoen agar (Kasvi, Roseto degli Abruzzi, Italy). Phenotypic identification of genera/species of *E. coli*, *Shigella*, and *Salmonella* was performed by biochemical tests [40]. Antimicrobial susceptibility tests were performed by the disk and strip-diffusion method, according to standards and guidelines from the Clinical and Laboratory Standards Institute (CLSI, 2019). Bacterial isolates were tested for antimicrobial resistance to the following antimicrobial agents: amikacin, gentamicin, tobramycin, ampicillin, ampicillin-sulbactam, amoxicillin-clavulanate, piperacillin-tazobactam, cefoxitin, cefotaxime, ceftazidime, cefepime, aztreonam, imipenem, meropenem, ertapenem, doxycycline, nitrofurantoin, ciprofloxacin, levofloxacin, ofloxacin, chloramphenicol, tetracycline, and trimethoprim-sulfamethoxazole (disks from CECON, São Paulo, Brazil; M.I.C. Evaluator Strips from Oxoid, Basingstoke, UK).

Two to four *E. coli* colonies from each specimen were subjected to two multiplex PCRs, as previously described [41]. PCR 1 assay contained the primer mix for detection of the *E. coli* attaching and effacing gene (*eae*), bundle*-*forming pilus gene (*bfp*), and anti-aggregation protein transporter gene (*aat*, previously known as CVD432). PCR 2 assay contained specific primers for thermolabile (*elt*) and thermostable toxin (*est*) genes, invasion plasmid-encoded antigen H gene (*ipaH*), and Shiga toxin genes (*stx1* and *stx2*). These assays allow identifying typical (*eae*^+^, *bfp*^+^) and atypical (*eae*^+^, *bfp*^−^, *stx1*^−^, *stx2*^−^) EPEC; typical enteroaggregative *E. coli* (*aat*^+^) and enterotoxigenic *E. coli* (*elt*^+^ and/or *est*^+^); *Shigella* or enteroinvasive *E. coli* (*ipaH*^+^); EHEC (*eae*^+^, *bfp*^−^, *stx1*^+^, and/or *stx2*^+^); and STEC *eae*^−^ (*eae*^−^, *bfp*^−^, *stx1*^+^, and/or *stx2*^+^). The reference strains enteroaggregative *E. coli* EAEC 042 (*aat*^+^), tEPEC E2342/69 (*bfp*^+^, *eae*^+^), enterotoxigenic *E. coli* H10407 (*st*^+^, *lt*^+^), EHEC EDL933 (*eae*^+^, *stx2*^+^), and entero-invasive *E. coli* EDL1284 (*ipaH*^+^) were included in the PCR assays as positive controls.

EHEC isolates were serotyped by tube agglutination using absorbed antisera for somatic antigens O1 to O183 and flagellar antigens H1 to H56. Somatic antigens O184, O185, O186, O187, and O188 were screened by multiplex PCR [40, 42].

### PFGE, MLST, and genetic characterization of virulence factors in EHEC

The clonal relationship of EHEC isolates was analyzed according to Durmaz et al. (2009) [43]. PFGE was performed after macrorestriction with *Xba*I in a CHEF-DR III system (Bio-Rad, USA) and analyzed using GelJ software [44] by the unweighted pair-group method with arithmetic mean (UPGMA) and Dice coefficient. Isolates were considered to belong to the same pulsotype if they shared at least 80% similarity in band patterns. The ST of the EHEC strain was characterized by MLST analysis after the sequencing of seven *E. coli* housekeeping genes (*adk*, *fumC*, *gyrB*, *icd*, *mdh*, *purA*, and *recA*), and comparison of data against the *E. coli* MLST database (http://enterobase.warwick.ac.uk/species/ecoli/search_strains), according to previous recommendations [45].

Virulence genes (*efa1*, *nleE*, *nleb*, *sem*, *pagC*, *terE*, *katP*, *ehxA*, *toxB*, *espP*, *iha*, *astA*, *sat*, *set1A*, and *chuA*) were investigated by PCR, as previously described [16, 46–49]. The *stx2* and *eae* genes were subtyped by Sanger sequencing on a Thermo Fisher Scientific ABI 3500 platform [50, 51].

### Detection and molecular characterization of gastroenteric viruses

Rotavirus and norovirus GI and GII were investigated in 24 symptomatic and 6 asymptomatic cases. Viral nucleic acids were purified from 140 μL of stool suspension (10% w/v) by an automatic nucleic acid extraction procedure using a QIAamp® Viral RNA Mini kit (QIAGEN, CA, USA) in a QIAcube® automated system (QIAGEN). Viruses were detected and quantified by using TaqMan®-based qPCR protocols, as previously described [52, 53]. Primers (COG1F and R; COG2F and R) and probes (RING1C and RING2) targeting ORF1/2 were used to detect norovirus GI and GII, respectively. For rotavirus detection, primers (NSP3F and R) and probe (NSP3p) targeting the conserved NSP3 gene were used. Primers targeting the 3′-end of ORF1 and 5′-end of ORF2 (Mon 431 and G2SKR), which generated a ∼ 557 bp amplicon, were used for molecular characterization of norovirus GII [24]. Sanger sequencing was performed using both forward and reverse primers with the BigDye™ Terminator v. 3.1 Cycle Sequencing Kit (Applied Biosystems, CA, USA), and reactions were run at the FIOCRUZ Institutional Sequencing Platform (PDTIS) on an ABI Prism 3730xl genetic analyzer (Applied Biosystems). Consensual sequences were obtained using Geneious prime (Biomatters Ltd., Auckland, New Zealand). Norovirus genotypes were firstly assigned using two norovirus typing tools (https://www.rivm.nl/mpf/typingtool/norovirus and https://norovirus.ng.philab.cdc.gov). Phylogenetic trees were constructed by the maximum-likelihood method and Kimura two-parameter model (2000 bootstrap replications for branch support) in MEGA X [54] using norovirus reference sequences obtained from the National Center for Biotechnology Information (NCBI) database. Norovirus GII nucleotide sequences were submitted to GenBank and assigned the following accession numbers: MT129134 to MT129138.

## Supplementary Information

**Additional file 1.**

## Data Availability

The sequencing data generated in this study were submitted to GenBank and assigned the following accession numbers: MT129134 to MT129138.

## Abbreviations

EHEC: Enterohemorrhagic *Escherichia coli*
STEC: Shiga toxin-producing *Escherichia coli*
HUS: Hemolytic uremic syndrome
HC: Hemorrhagic colitis
MLST: Multilocus sequence typing
ST: Sequence type
SLV: Single-locus variant
LEE: Locus of enterocyte effacement
PFGE: Pulsed-field gel electrophoresis
EPEC: Enteropathogenic *Escherichia coli*
