# Supplementary material for: EHEC O111:H8 strain and norovirus GII.4 Sydney [P16] causing an outbreak in a daycare center, Brazil, 2019
Source: BMC Microbiol. 2021 Mar 29;21:95. doi: 10.1186/s12866-021-02161-x (PMC8008580; doi:10.1186/s12866-021-02161-x)
Supplement: Supplementary file 1 — Additional file 1. [file 12866_2021_2161_MOESM1_ESM.docx]

**EHEC O111:H8 strain and norovirus GII.4 Sydney[P16] causing an outbreak** **in a daycare center, Brazil, 2019**

Liliana Cruz Spano*^1†^, Caroline Gastaldi Guerrieri^1†^, Lays Paula Bondi Volpini^1^, Ricardo Pinto Schuenck^1^, Jaqueline Pegoretti Goulart^2^, Elizabeth Boina^2^, Celia Regina Nascimento Recco^3^, Rodrigo Ribeiro-Rodrigues^1,2^, Luís Fernando dos Santos^4†^, and Tulio Machado Fumian^5†^

^1^Department of Pathology, Health Sciences Center, Federal University of Espírito Santo, Vitória, Brazil

^2^State Health Secretariat, Central Public Health Laboratory, Vitoria, Espírito Santo, Brazil

^3^Municipal Health Secretariat, Epidemiology Service, Vila Velha, Espírito Santo, Brazil

^4^Adolfo Lutz Institute, Centre of Bacteriology, National Reference Laboratory for *Escherichia coli* Enteric Infections, São Paulo, Brazil

^5^Laboratory of Comparative and Environmental Virology, Oswaldo Cruz Institute, Fiocruz, Rio de Janeiro, Brazil

*** Correspondence:** liliana.spano@ufes.br (L. C. Spano)

^†^ **These authors contributed equally to this article**

**Supplementary Table**: Clinical and demograhic data of symptomatic individuals affected in the outbreak of diarrhea, hemorrhagic colitis, and hemolytic uremic syndrome

| **Patient** | **Age** | **Daycare** | **Occupation** | **Class** | **Date*** | **Diarrhea** | **Mucus** | **Blood** | **Vomit** | **Fever** | **Seizure** | **Hematuria** | **Hospitalization** | **EHEC** | **Norovirus** |
| --- | --- | --- | --- | --- | --- | --- | --- | --- | --- | --- | --- | --- | --- | --- | --- |
| 1 | 2y1m | Yes | Student | 2 | 03/15 | + | + | + | - | + | - | + | + | - | - |
| 2 | 3y | Yes | Student | 3B | 03/15 | - | - | - | + | + | - | - | - | - | - |
| 3 | 1y6m | Yes | Student | Baby | 03/16 | + | - | - | - | + | - | - | + | - | + |
| 4 | 2y5m | Yes | Student | 2 | 03/18 | + | + | + | + | - | + | - | + | - | - |
| 5 | 3y1m | Yes | Student | 3A | 03/18 | + | + | + | + | + | - | + | + | - | + |
| 6 | 2y9m | Yes | Student | 2 | 03/18 | + | + | - | - | - | - | - | - | + | + |
| 7 | 27y9m | Yes | Assistant | 3A | 03/18 | + | - | - | - | - | - | - | - | - | + |
| 8 | 2y1m | Yes | Student | 2 | 03/19 | + | + | - | - | - | - | - | - | - | - |
| 9 | 1y | Yes | Student | Baby | 03/19 | + | - | - | - | - | - | - | - | - | - |
| 10 | 5y | Yes | Student | 5 | 03/21 | + | - | - | - | - | - | - | - | - | NA |
| 11 | 2y6m | Yes | Student | 2 | 03/22 | + | - | - | + | - | - | - | + | + | - |
| 12 | 37y7m | Yes | Teacher | 2 | 03/23 | + | - | - | - | - | - | - | - | - | - |
| 13 | 7m | Yes | Student | Baby | 03/23 | + | - | - | + | + | - | - | - | - | + |
| 14 | 55y | Yes | Other | NA | 03/24 | + | - | + | + | - | - | - | - | - | + |
| 15 | 6y | No | Family | NA | 03/24 | + | - | - | + | + | - | - | - | - | - |
| 16 | 7y7m | Yes | Student | 5 | 03/25 | + | - | - | + | + | - | - | - | - | - |
| 17 | 34y | Yes | Other | NA | 03/25 | + | - | - | - | - | - | - | - | - | - |
| 18 | 41y6m | Yes | Other | NA | 03/26 | + | - | - | - | + | - | - | - | - | NA |
| 19 | 1y1m | Yes | Student | Baby | 03/26 | + | - | - | - | - | - | - | + | - | NA |
| 20 | 2y1m | No | Family | NA | 03/27 | + | - | - | + | - | - | - | + | - | + |
| 21 | 11m | Yes | Student | Baby | 03/28 | + | - | - | - | - | - | - | - | - | + |
| 22 | 35y4m | Yes | Teacher | 3A | 03/29 | + | - | - | - | - | - | - | - | - | - |
| 23 | 65y | No | Family | NA | 03/29 | + | - | - | - | - | - | - | - | - | + |
| 24 | 59y | No | Family | NA | 03/29 | + | - | - | + | - | - | - | - | - | + |
| 25 | 39y | No | Family | NA | 03/30 | + | - | - | + | + | - | - | - | - | + |
| 26 | 7m | No | Family | NA | 04/01 | + | - | - | + | + | - | - | - | - | - |
| 27 | 28y | No | Nurse | NA | 04/07 | + | - | + | - | + | - | - | - | - | - |

at a daycare center in southeastern Brazil

*Symptom onset date; y= years, m= months, NA= Not applicable
